# Need for Medication Review and Deprescribing in People Living with HIV: A Longitudinal Observational Study

**DOI:** 10.3390/ph19091383

**Published:** 2026-09-01

**Authors:** Emanuela De Bellis, Danilo Donnarumma, Annarita Pagano, Ines Mazza, Bruno Charlier, Simona Madonia, Alfonso Masullo, Mirella Onofrio, Valentina Fortunato, Anna Maria Spera, Federica Campana, Gianluigi Franci, Flora Salzano, Graziamaria Corbi, Amelia Filippelli, Pasquale Pagliano, Valeria Conti

**Affiliations:** 1 PhD School “Clinical and Translational Oncology (CTO)”, Scuola Superiore Meridionale, University of Naples “Federico II”, 80138 Naples, Italy; emanuela.debellis@unina.it (E.D.B.); d.donnarumma@ssmeridionale.it (D.D.); 2 Postgraduate School of Clinical Pharmacology and Toxicology, University of Salerno, 84081 Baronissi, Italy; annpagano@unisa.it (A.P.); imazza@unisa.it (I.M.); 3 Department of Medicine, Surgery and Dentistry “Scuola Medica Salernitana”, University of Salerno, 84081 Baronissi, Italy; bcharlier@unisa.it (B.C.); gfranci@unisa.it (G.F.); flsalzano@unisa.it (F.S.); afilippelli@unisa.it (A.F.); vconti@unisa.it (V.C.); 4 Clinical Pharmacology Unit, San Giovanni di Dio e Ruggi d’Aragona University Hospital, 84131 Salerno, Italy; 5 Infectious Diseases Unit, San Giovanni di Dio e Ruggi d’Aragona University Hospital, 84131 Salerno, Italy; simona.madonia@sangiovannieruggi.it (S.M.); alfonso.masullo@sangiovannieruggi.it (A.M.); mirella.onofrio@sangiovannieruggi.it (M.O.); valentina.fortunato@sangiovannieruggi.it (V.F.); annamaria.spera@sangiovannieruggi.it (A.M.S.); 6 Clinical Infectious Diseases Unit, San Giovanni di Dio e Ruggi d’Aragona University Hospital, 84131 Salerno, Italy; 7 Department of Human, Philosophical and Formation Sciences, University of Salerno, 84084 Fisciano, Italy; fcampana@unisa.it; 8 Microbiology and Virology Unit, San Giovanni di Dio e Ruggi D’Aragona University Hospital, 84131 Salerno, Italy; 9 Department of Translational Medical Sciences, University of Naples “Federico II”, 80131 Naples, Italy; graziamaria.corbi@unina.it

**Keywords:** polypharmacy, viremia, drug–drug interactions, drug–supplement interactions, drug resistance, natural health products, adverse events, highly active antiretroviral therapy, drug toxicity, drug interaction checkers

## Abstract

**Background/Objectives:** People Living with HIV (PLWH) often have comorbidities require polypharmacy, increasing the risk of adverse events (AEs) associated with drug–drug/drug–supplement interactions (DDIs/DSIs). However, DDIs and DSIs, including those involving antiretrovirals (ARVs), and their related effects have not been exhaustively assessed. This study aimed to determine, in HIV patients on HAART who are taking multiple medications, the prevalence of DDIs and DSIs involving both ARVs and non-ARV co-medications, and their clinical relevance and consequences. **Methods**: In this longitudinal observational study, PLWH receiving HAART were consecutively enrolled at the University Hospital of Salerno and followed up during scheduled visits. Demographic, clinical, and pharmacological data were collected. DDIs/DSIs were identified using Drugs.com, Lexicomp, and the Liverpool HIV Interaction Checker. Interactions classified as major (Drugs.com), consider therapy modification or avoid combination (Lexicomp), and do not coadminister (Liverpool) were deemed highly clinically relevant. This assessment was supplemented with review of SmPCs and the available literature. **Results**: Among 84 PLWH, 229 interactions were identified, and 37/229 (16.2%) were highly clinically relevant. Eight patients (9.5%) experienced AEs predicted by the tools. Four were DDIs: cobicistat/alprazolam associated with sedation and dyspnoea, rilpivirine/hydroxychloroquine with QT prolongation, bictegravir/ursodeoxycholic acid with rising viremia, and atorvastatin/fenofibrate with myalgia. Four DSIs between bictegravir and supplements containing divalent ions have been associated with increasing viremia. After medication review, alprazolam was replaced with zolpidem, rilpivirine-based HAART with bictegravir/emtricitabine/tenofovir alafenamide, and atorvastatin/fenofibrate with simvastatin/ezetimibe, and ursodeoxycholic acid and supplements were discontinued. These interventions were temporally associated with clinical improvement of interaction-related AEs and good virological control. **Conclusions**: This study highlights the importance of predicting DDIs/DSIs in PLWH undergoing polypharmacy. Medication review supports clinical decision-making, preventing avoidable adverse outcomes.

## 1. Introduction

Highly Active Antiretroviral Therapy (HAART) has significantly increased the life expectancy of People Living with HIV (PLWH), transforming Human Immunodeficiency Virus (HIV) infection from a fatal to a chronic condition [1]. On the other hand, there has been an increase in cases involving comorbidities [2,3,4,5], which are expected to affect up to 70% of PLWH by 2030 [4,6,7]. Some of these are due to chronic inflammation linked to the persistent presence of the virus, which accelerates the onset of age-related diseases [8,9] whilst others are related to both the same inflammatory pathways in the central nervous system and the psychosocial factors associated with HIV illness [10]. For these reasons, PLWH take additional medicines alongside HAART and often need a polypharmacy regimen, increasing the risk of iatrogenic toxicity [11]. Indeed, polypharmacy is strongly associated with an increased risk of adverse events (AEs) with a direct impact on medication adherence [12] and healthcare resources, such as hospitalization and length of stay, leading to a significant increase in healthcare costs [13,14]. Furthermore, the socio-economic impact of HIV is greatly amplified by factors such as unemployment, low educational attainment, and social isolation, which are strongly associated with increased multimorbidity and poor medication adherence and persistence, particularly among ethnic minorities [4,15,16,17].

Poor adherence to HAART is one of the main factors leading to the development of viral resistance [17]. Indeed, irregular intake of antiviral drugs leads to fluctuations in plasma drug levels, creating the conditions for the selection of viruses resistant to the antivirals that make up HAART [18]. Available data indicate that even moderate reductions in adherence to HAART are associated with a significant acceleration of intra-host selection of resistant variants and community transmission of drug-resistant HIV strains [19,20]. AEs may be caused or exacerbated by pharmacological interactions involving drugs (drug–drug interactions, DDIs) or drugs and supplementation (drug–supplement interactions, DSI) with natural health products (NHPs). The simultaneous intake of two or more pharmacologically active molecules can lead to a mutual alteration of their pharmacodynamic and pharmacokinetic parameters [21].

Among the agents included in the HAART regimens, protease inhibitors (PIs), followed by non-nucleoside reverse transcriptase inhibitors (NNRTIs) and integrase inhibitors (INIs), are involved in clinically relevant DDIs. PIs, and their boosting agents, such as ritonavir and cobicistat, often used to slow down the enzymatic activity of CYP450 isoforms that metabolize co-formulated antivirals, also inhibit the metabolism of other drugs used to treat comorbidities, including statins, benzodiazepines (BDZs), hypoglycaemic agents, and proton pump inhibitors [2,3,5,22], increasing the risk of DDI- and DSI-related AEs. In addition, HIV patients frequently are treated with drugs that may accelerate the metabolism of antiretrovirals (ARVs), such as INIs, to the extent that their therapeutic effect is reduced, leading to virological failure.

However, DDIs and DSIs, including those involving ARVs, have mostly been the subject of retrospective observational studies [22,23,24], which have been unable to assess the associated clinical outcomes. Some studies have examined interactions and their potential impact on safety and efficacy only between specific ARVs and specific medicines [25,26,27], or by not considering interactions between non-HIV co-medications and failing to perform full recognition of patients’ polypharmacy [28], thereby limiting personalized and effective management in clinical practice. From a patient-centred perspective, Medication Reviews and Deprescribing (MRDP) represents a key strategy for reducing Potentially Inappropriate Medication (PIM) and improving drug safety in patients receiving multiple medications [29].

This longitudinal observational study aimed to determine, in HIV patients on HAART who are taking multiple medications, the prevalence of DDIs and DSIs involving both ARVs and non-ARV co-medications, as well as their clinical relevance and consequences.

## 2. Results

Eighty-four adult patients of both sexes receiving HAART were enrolled.

The main characteristics of the study population are shown in Table 1. Figure S1 shows the study flow diagram (Figure S1).

Considering all the medications taken by the patients, the drug interaction checkers identified 229 interactions. Of these, 37 (16.2%) were deemed highly clinically relevant: 28 were identified by Drugs.com [30], 22 by Lexicomp [31], and 2 by Liverpool HIV Interactions [32]. In twenty-four cases (64.9%), the drugs involved were ARVs. Among them, the drugs most frequently involved were bictegravir (*n* = 6, 25.0%), cobicistat (*n* = 5, 20.8%), darunavir (*n* = 4, 16.7%) and tenofovir alafenamide (*n* = 3, 12.5%). Multivitamins with minerals were involved in the 10,8% of cases (*n* = 4), followed by pantoprazole (*n* = 3, 8.11%) (Tables S1 and S2).

During the study, eight patients (9.5%) experienced AEs; four of these were related to DDIs and four to DSIs. MRDP was requested by infectious disease specialists and was carried out by clinical pharmacologists, who provided a report containing possible recommendations and suggestions regarding changes to treatment—including dose adjustment—or clinical and therapeutic drug monitoring (TDM). The final decision was always up to the infectious disease specialist who requested the MDRP.

This service, available on request from the Clinical Pharmacology Unit at Salerno University Hospital, aims to identify potential drug-related problems, including drug duplications, drug omissions, contraindications and risks arising from pharmacological interactions. The Unit has developed a specific form on which clinical, demographic and biometric data, and lifestyle information, as well as details of any pharmacogenetic tests or TDM that have already been carried out or requested, are recorded. In addition, full recognition is carried out of the drugs and supplementation taken by the patient, including details of the active ingredient and trade name, dosage and route of administration. DDIs and DSIs are searched by consulting the available literature and the SmPCs for each listed medicine, and by using different drug interaction checkers.

The list of drugs and supplements used by these patients is shown in Table 2 and Table 3. A description of these clinical cases is provided below. Table 4 summarizes patient clinical characteristics together with the severity of DDIs/DSIs and the proposed mechanism, as well as clinical outcomes following MRDP (Table 4).

Patient 1 is a 67-year-old man who was diagnosed with HIV in 2012 in the United States (US) and then treated at the University Hospital of Salerno since 2022. Although the clinical records from the US were fragmentary, the patient was documented to have a history of anxiety–depressive disorder, atrial fibrillation (AF) and heart failure. At the first visit in Salerno (baseline), laboratory tests showed an undetectable HIV viral load and a CD4^+^ T-cell count ranging was above 500 and 800 cells/mm^3^, indicating a good virological control. However, the patient reported marked episodes of drowsiness.

At the first FU, the patient provided a further medical report relating to a previous episode of bronchopneumonia. He reported that he had completed a course of treatment with antibiotics (unspecified) and was currently being treated with umeclidinium bromide and fluticasone/vilanterol as supportive bronchodilator therapy. Following this treatment, the clinical picture improved, but dyspnoea persisted. In addition, furosemide had been replaced with hydrochlorothiazide and rosuvastatin with evolocumab due to poor lipid profile control (Table 2).

At the second FU, the patient reported that he had completed his course of bronchodilator treatment. However, his condition had deteriorated significantly, to the point where he required assistance from a caregiver for the simplest daily activities. In particular, his shortness of breath had worsened considerably. Seven clinically relevant DDIs were identified, three by Drugs.com [30] and four by Lexicomp [31]. Both tools identified a clinically significant DDI involving alprazolam and cobicistat. Specifically, the tools reported that potent CYP3A4 inhibitors, such as cobicistat, can markedly increase the plasma exposure of BDZ, which are extensively metabolized by this isoenzyme, such as alprazolam, leading to possible excessive sedation and respiratory depression. Therefore, concomitant use of BDZ with potent CYP3A4 inhibitors is contraindicated. The cobicistat-alprazolam DDI is also described in the summary of product characteristic (SmPC) of both medicinal products. In particular, the darunavir/cobicistat SmPC states that cobicistat is a selective inhibitor of the CYP3A subfamily of cytochrome P450 enzymes and that this cobicistat-mediated metabolic inhibition leads to increased systemic exposure of CYP3A substrates [33]. Similarly, the Italian SmPC of alprazolam states that agents inhibiting CYP3A4 may increase plasma concentrations of alprazolam and that symptoms of overdose include, among others, drowsiness and respiratory depression [34]. Based on these data, the clinical pharmacology team recommended replacing alprazolam with zolpidem, the metabolism of which is less dependent on the activity of the CYP3A enzyme [35]. It is important to note that neither the tools nor the zolpidem SmPC [35] identified any clinically significant DDI between zolpidem and cobicistat, or between zolpidem and the other medications taken by the patient. Furthermore, paroxetine was deprescribed. Clinical pharmacologists met with the patient 2 and 4 months after these therapeutic changes during scheduled visits. The patient denied dyspnoea and reported no longer needing assistance, having regained some independence in daily activities. In addition, infectious disease specialists simplified HAART by replacing the treatment with darunavir/cobicistat, emtricitabine/tenofovir disoproxil, and dolutegravir with a regimen with bictegravir/emtricitabine/tenofovir alafenamide (Table 2).

Patient 2 is a 64-year-old man diagnosed with HIV in 2000. The patient had severe left ventricular systolic dysfunction requiring cardioverter-defibrillator implantation. Additional cardiovascular conditions included moderate to severe mitral regurgitation, chronic AF and arterial hypertension. Based on a cardiology report from February 2024, the patient had prolonged QTc. At that time, therapy with amiodarone and folic acid was discontinued. The patient also suffered from chronic obstructive pulmonary disease (COPD), ankle arthritis, and had a history of infective endocarditis involving an intracardiac electronic device (December 2019). The clinical history included accessory pathway ablation for Wolff- Parkinson-White syndrome (1994), stage III chronic kidney disease, left inguinal hernioplasty, bilateral cataract surgery (right eye in 2021 and left eye in 2022) and a history of syphilis. The list of drugs is shown in Table 2. Over the years, the patient had maintained good virological and immunological control. In March 2025, the CD4+ T-cell count was 500 cells/mm^3^. After admission for urinary septic shock due to extended-spectrum beta-lactamase-producing Escherichia coli, complicated by acute renal failure and ECG abnormalities (particularly QTc prolongation = 510 ms), the patient was referred to the Clinical Pharmacology Unit for medication review and deprescribing. On that occasion, the patient also reported long-standing leg pain and marked asthenia.

Clinical pharmacologists identified two clinically relevant DDIs through Drugs.com [30]. One of these concerned a warning to avoid hydroxychloroquine in combination with rilpivirin, another agent associated with QTc prolongation, particularly in patients with baseline QTc prolongation or congenital long QTc syndrome.

This possibility also emerged from an analysis of the hydroxychloroquine SmPC, which states that hydroxychloroquine should be used with caution when administered concomitantly with agents that prolong the QTc interval, as there is an increased risk of ventricular arrhythmias [36]. The SmPC of emtricitabine/rilpivirine/tenofovir alafenamide also states that, although rilpivirine at the recommended dosage does not appear to have a clinically significant effect on the QTc interval, caution is advised when administered concomitantly with medicines known to carry a risk of torsades de pointes [37]. Based on this evidence, the HAART regimen was replaced with another (i.e., bictegravir/emtricitabine/tenofovir alafenamide) that is not associated with significant cardiac effects. Six months later, optimal virological control was maintained. Ten months later, the patient was admitted to the hospital with bacterial pneumonia, which exacerbated the underlying heart condition. Consequently, vitamin B12 (1000 mcg daily), folic acid (5 mg daily), vitamin D3 (10,000 IU daily) and cortisone acetate (25 mg daily) were administered, while the HAART regimen remained unchanged and the viral load remained undetectable.

Patient 3 is a 38-year-old man with a history of HIV, who also suffers from hypercholesterolaemia, arterial hypertension and AF. Despite adherence to HAART, the patient reported a steady rise in viral load to 3490 copies/mL. No HAART resistance was detected. The assessment of potential DDIs or DSIs identified a DDI between bictegravir/emtricitabine/tenofovir alafenamide and ursodeoxycholic acid identified by Liverpool HIV Interactions [32] as “potential interaction”. Although only pharmacological interactions defined as clinically relevant in the study population were included in the overall analysis, the clinical case concerning ursodeoxycholic acid and bictegravir was described because it is an example of medication review and deprescribing in clinical practice and clearly demonstrates the usefulness of this process.

According to Liverpool HIV Interactions, ursodeoxycholic acid may reduce plasma concentrations of bictegravir through a potential induction effect on CYP450 enzymes, particularly CYP3A, which, together with UGT1A1, plays an important role in bictegravir metabolism. This report is consistent with the SmPC of bictegravir/emtricitabine/tenofovir alafenamide, which states that concomitant administration of bictegravir and medicinal products that are potential inducers of both CYP3A and UGT1A1 is contraindicated due to a reduction in plasma concentrations of bictegravir, resulting in a loss of therapeutic effect [38].

**Figure 1:**
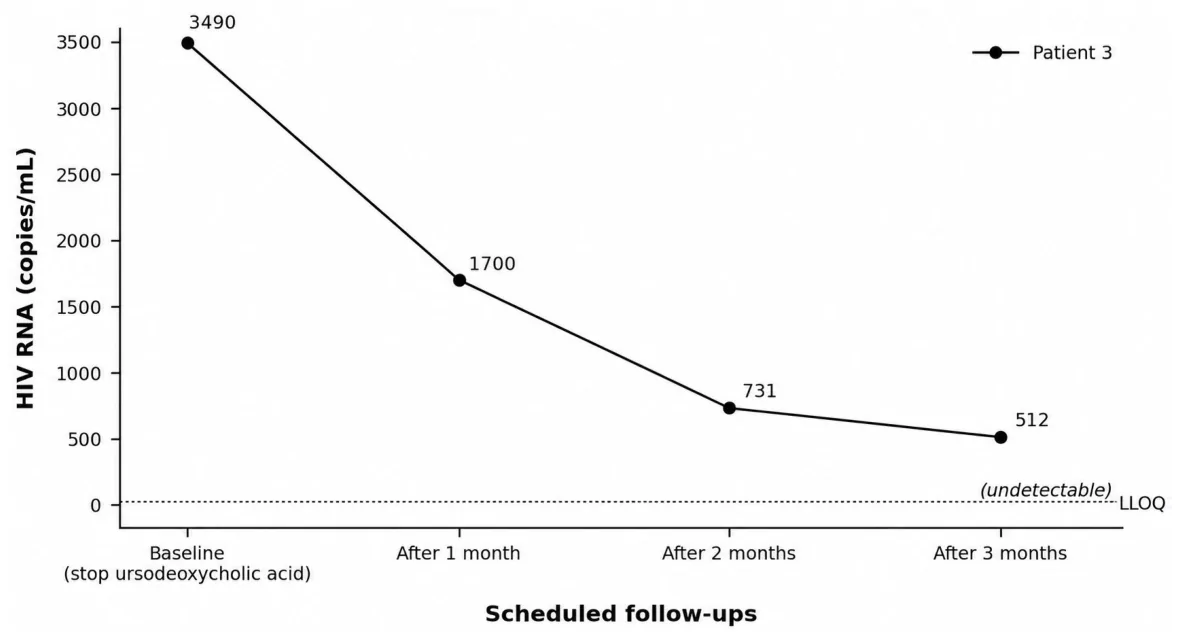
Viremia of Patient 3 at the time of enrolment and at three scheduled follow-ups.

Furthermore, the SmPC of ursodeoxycholic acid reports in vitro evidence indicating that this drug may induce CYP3A [39]. None of the other concomitant drugs had an inductive effect on UGT1A1. Based on the results of blood tests, which showed that the patient no longer required choleretic or hepatoprotective treatment, it was decided to deprescribe ursodeoxycholic acid. Viremia was measured three times during three months following the change in treatment, showing a significant progressive decrease as follows, 1700 copies/mL, 731 copies/mL and 512 copies/mL, although virological suppression was not achieved during the observed follow-up period (Figure 1).

Patient 4, who contracted HIV infection in 1989, also suffered from arterial hypertension, hypercholesterolemia, and anxiety–depression disorder. At the time of enrolment, the patient reported marked episodes of asthenia accompanied by muscle weakness and myalgia.

Drugs.com [30] identified a clinically relevant DDI between atorvastatin and fenofibrate associated with potential severe muscle toxicity. The SmPC of both fenofibrate and atorvastatin states that the risk of severe muscle toxicity increases if a fibrate is used in combination with statins or other fibrates [40,41]. Moreover, atorvastatin SmPC recommends using the lowest possible dose of this statin if concomitant administration cannot be avoided [41].

**Figure 2:**
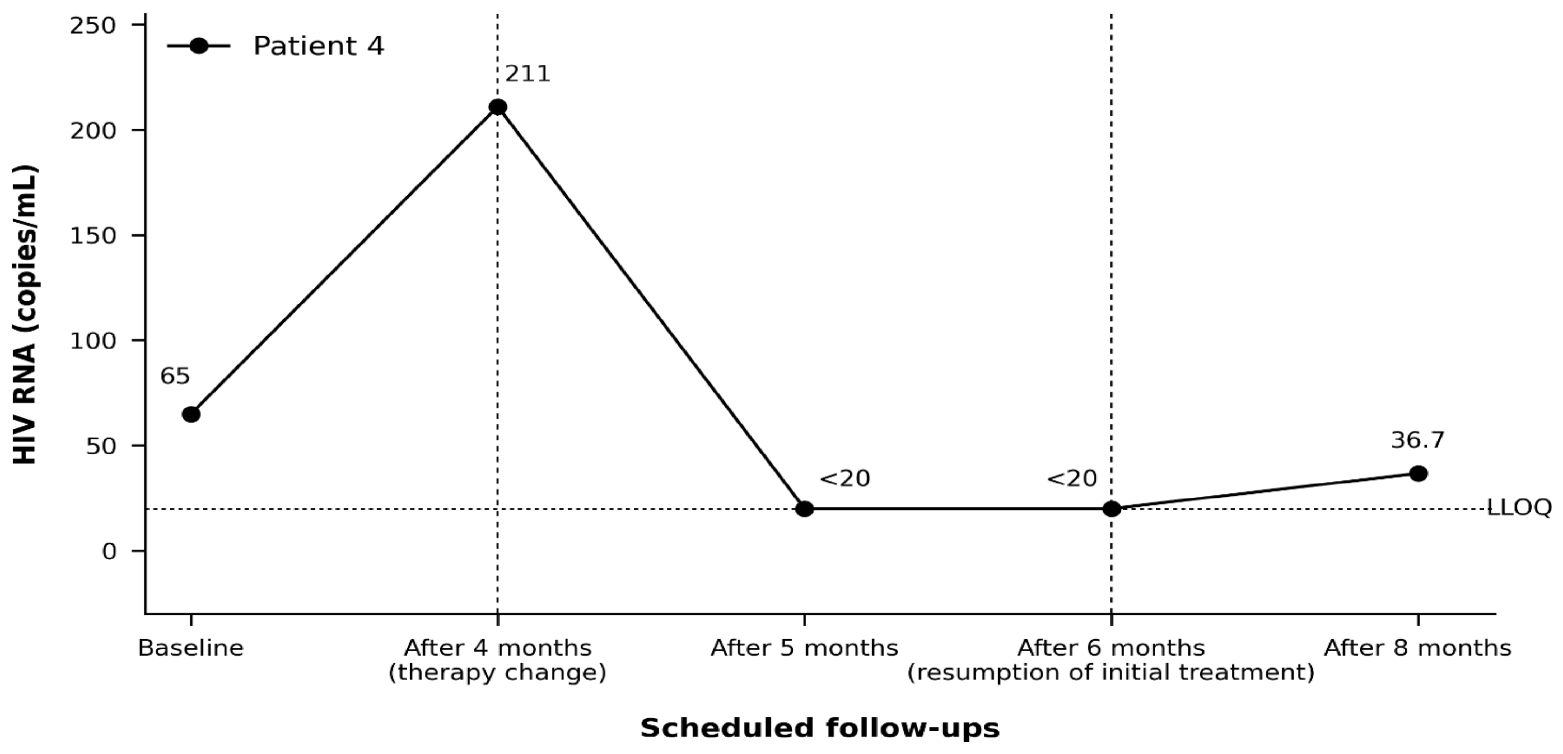
Viremia of Patient 4 at the time of enrolment and during the following months. Abbreviations: LLOQ, lower limit of quantification.

Furthermore, the patient had fluctuating viral load, which had reached 211 copies/mL whilst taking 20 mg atorvastatin and 145 mg fenofibrate daily. Given the muscle toxicity in the presence of a satisfactory lipid profile, lipid-lowering therapy was switched to simvastatin/ezetimibe 20/10 mg. Following this change, viral load fell to <20 copies/mL and the toxicity resolved. Unfortunately, due to an increase in LDL and triglycerides, the patient’s general practitioner reinstated the previous therapy with 20 mg atorvastatin plus fenofibrate. Viral load rose again to 36.7 copies/mL (Figure 2). This is a cause for concern because, although some evidence suggests that fibrates may act as inducers of CYP3A4 and UGT1A1 [42], the available data are too limited to justify eventual changes to HAART.

Fluctuating viral load has also been observed in patients taking NHPs. In these subjects (three men and one woman), this could be attributed to DSI.

The polypharmacy administered to these patients is shown in Table 3.

Analysis of medical records revealed that Patient 5 suffered from COPD, chronic hepatitis C, hypercholesterolemia, and arterial hypertension. Patient 6 suffered from hypercholesterolemia, hypertension, and unstable angina. Patient 7 had arthritis and fibromyalgia. Patient 8 suffered from hypercholesterolemia and arterial hypertension.

**Figure 3:**
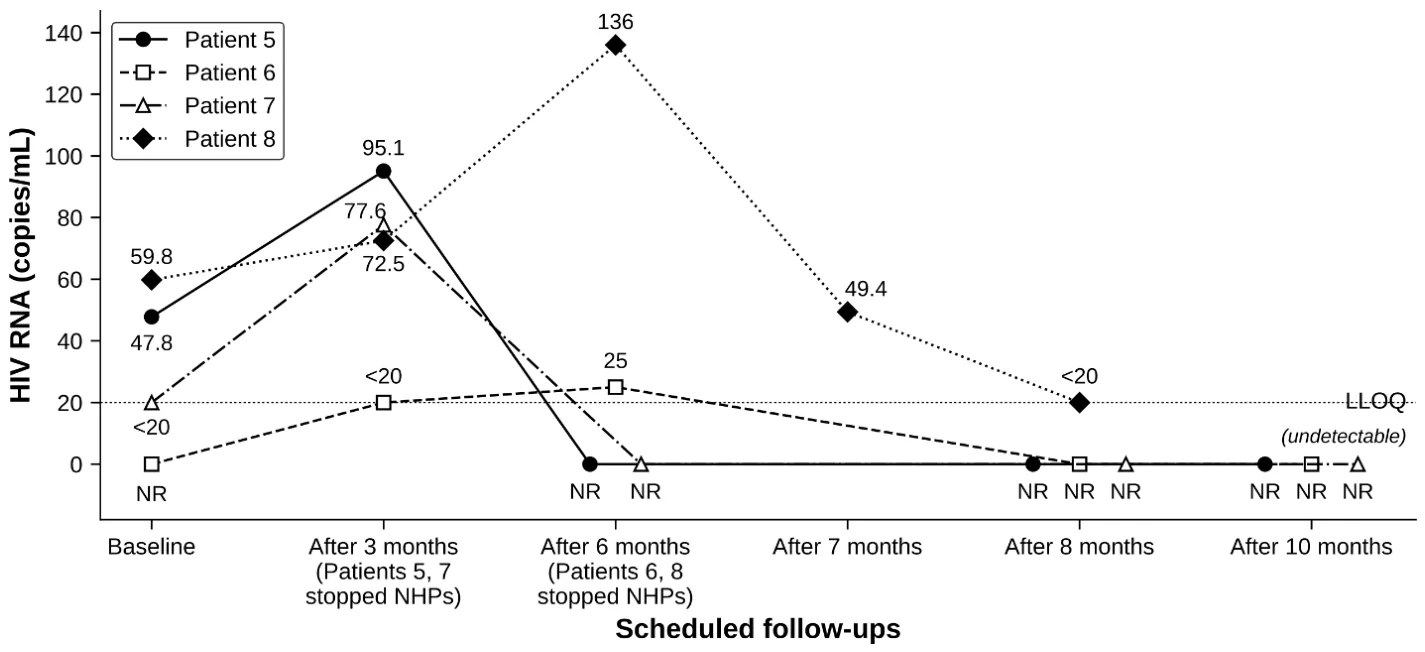
Viremia of Patients 5, 6, 7 and 8 measured at baseline and at scheduled follow-ups. Abbreviations: LLOQ, lower limit of quantification.

Patients 6 and 8 showed progressive increase in viremia (although this remained at low levels) from baseline to the second FU (Patient 6: baseline undetectable, first FU < 20 copies/mL, second FU 25 copies/mL; Patient 8: baseline 59.8 copies/mL, first FU 72.5 copies/mL, second FU 136 copies/mL). Patients 5 and 7 exhibited fluctuating viremias across the three assessment time points (Patient 5: baseline 47.8 copies/mL, first FU 95.1 copies/mL, second FU undetectable; Patient 7: baseline < 20 copies/mL, first FU 77.6 copies/mL, second FU undetectable) (Figure 3).

All patients were receiving the same HAART regimen (i.e., bictegravir/emtricitabine/tenofovir alafenamide) and were concomitantly taking supplements containing polyvalent cations, specifically magnesium (Patients 5–7) and calcium (Patient 8). All patients reported frequently using these products to relieve symptoms of fatigue and weakness, particularly during the hottest periods of the year. These supplement formulations are typically marketed in packs covering treatment durations ranging from one to five weeks. Importantly, all patients reported daily use of these NPHs. However, it was not possible to determine the time interval between taking these supplements and the ARVs. The exact marketed NHP was informed by two out of four patients. Therefore, it was possible to establish the amount of polyvalent cations in Patient 6 (75 mg magnesium) and Patient 8 (136 mg calcium carbonate). In addition, Patient 8 reported using the supplement every evening to treat heartburn due to gastroesophageal reflux. In addition, when the heartburn became unbearable, the patient reported occasionally taking two 20 mg pantoprazole per day instead of one, contrary to the specialist’s prescription.

After excluding poor medication adherence to HAART, a systematic evaluation of potential DDI/DSI was performed. Since supplements are categorized differently by drug interaction checkers, we selected the term “multivitamins with minerals” for Drugs.com [30] and Lexicomp [31], and “magnesium supplements” and “calcium supplements” for Liverpool HIV Drugs Interaction [32]. The analysis identified one highly clinically relevant DSI for each patient (identified by Drugs.com), and two highly clinically relevant DSIs were identified for Patient 5 (both by Drugs.com) and for Patient 6 (one by Drugs.com and one by Lexicomp).

In all four cases, Drugs.com reported the same highly clinically relevant interaction between bictegravir and “multivitamins with minerals”. According to this tool, concomitant use of INIs and supplements containing polyvalent cations such as aluminum, magnesium, and calcium may reduce bioavailability of the antiretroviral agent. Although Drugs.com indicates that the exact mechanism of this interaction has not yet been fully elucidated, evidence from scientific literature suggests that INIs bind magnesium ions at the active site of HIV integrase. Consequently, taking supplements containing polyvalent cations could, by promoting the binding of the drug to these ions, reduce its binding to viral analogues, thereby compromising its antiviral efficacy [43,44]. Furthermore, the potential interaction between bictegravir/emtricitabine/tenofovir alafenamide and antacids and supplements is reported in the SmPC of this antiretroviral agent, as shown in Table 1 of Section 4.5: “Interactions with other medicines and other forms of interaction” [38]. In particular, when it is necessary to use supplements containing divalent cations, it is recommended to take ARVs at least 2 h apart to minimize the risk of treatment failure. Once this DSI had been identified as a possible cause of fluctuations in viral load, clinical pharmacologists suggested deprescribing NHPs. After discontinuing supplement use, patients’ viremia was monitored through routine blood tests. As shown in Figure 3, viremia in Patients 5 and 7, which was already undetectable at the first FU, remained unchanged 4 and 8 months after the second FU. Viremia of Patient 6 reached an undetectable level 4 months after discontinuing the supplement and remained undetectable after 8 months. Viremia of Patient 8, monitored five times, decreased significantly to 49.4 copies/mL two months after discontinuing NHPs, and subsequently fell below 20 copies/mL after 4 months (Figure 3). To be honest, these results do not prove that the combination of bictegravir and polyvalent cations can be considered one of the main causes of viral fluctuations in HIV patients. However, also considering the warning issued by Drugs.com, this interaction could be one of the interfering factors to be taken into account.

All AEs related to DDIs or DSIs described here have been reported to the Italian Medicines Agency (AIFA) via the pharmacovigilance portal.

## 3. Discussion

Since the introduction of HAART, the average life expectancy of HIV patients has increased significantly [45]. On the other hand, the management of HIV patients is now extremely complex because it requires addressing issues related to transient clinical conditions and comorbidities, as well as the occurrence of AEs associated with HAART regimens [4].

We found a 79% prevalence of comorbidities consistent with other studies enrolling even older patients [45]. The rather low mean age (54.6 years) of our study population may be the reason for the lack of evidence of comorbidities such as bone disease and dementia, which are instead reported in other studies [45].

Conversely, in line with literature data, we found that hypertension and dyslipidemia were the most frequent comorbidities followed by anxiety and depressive disorders and type II diabetes [46,47,48,49].

Multimorbidity leads to the prescription of multiple medications, each of which has the potential for iatrogenic toxicity, as well as risks from possible drug interactions.

However, the prevalence of polypharmacy among HIV patients varies widely, especially depending on the age and whether agents belonging to HAART regimens are included in the count [45,50,51].

Including ARVs, we found a 69% prevalence of polypharmacy, while excluding these drugs, the prevalence drops to 20%.

ARVs, particularly PIs and INIs, may be involved in DDIs or DSIs, leading to the occurrence of toxicity and decrease in efficacy, increasing the risk of virological failure [3,52,53].

Cardiovascular risk is a major concern in PLWH, and drugs such as statins and antiarrhythmics may be involved in DDIs with potentially dangerous consequences [54]. In Patient 4, the onset of a toxic synergy was observed, attributable to the concomitant use of 20 mg of atorvastatin and fenofibrate. This combination is in fact considered a probable cause of asthenia and myalgia, which are AEs associated with the use of both statins and fibrates, as reported in the SmPCs of both medicines. In addition, due to the viral blip in the absence of HAART resistance, a literature search was performed. Although based on limited evidence, a possible fenofibrate-induced effect on CYP3A4 and UGT1A1 was found, which could lead to reduced plasma exposure to bictegravir [38,55,56].

It is also necessary to closely monitor the increased cardiovascular risk when ARVs are taken alongside non-cardiovascular medicines. As evidence of this, in Patient 2, concomitant administration of hydroxychloroquine and rilpivirine was likely associated with QTc prolongation, a condition already present one year before the patient was enrolled in this study. The patient’s cardiologist had discontinued the antiarrhythmic drug amiodarone without considering that rilpivirine and hydroxychloroquine can also lead to QTc prolongation and that their combination may increase the risk of toxic synergy [57,58].

These clinical cases, as highlighted in other studies [59], emphasizes the need for an interdisciplinary approach to integrate pharmacological data with patient characteristics into the clinical decision-making process in daily clinical practice.

Neuropsychiatric disorders are also reported to be particularly prevalent among PLWH [46,47]. We found a rather low prevalence of anxiety and depressive disorders (8.7%), probably due to missed diagnosis.

Drugs used to treat these disorders are often influenced by the inhibitory effect of PIs. For example, co-administration of pharmacokinetic boosters may prolong BDZ effects [57]. Adverse events potentially related to DDI involving BDZ are revealed by the case of Patient 1, in which DDI with alprazolam/cobicistat may have caused or exacerbated episodes of drowsiness and shortness of breath, as also reported in the alprazolam SmPC [34]. Although literature data are scarce, it has already been suggested that BDZ, such as midazolam and alprazolam, in patients taking PI, may cause prolonged sedation, delayed recovery, and increased length of stay [57,60,61]. Other BDZ-related toxic effects, such as worsening neurocognitive performance, should be monitored in HIV patients [60,61].

Patient 3 had elevated viremia despite medication adherence and the absence of HAART-resistance. The MRDP highlighted a possible role for ursodeoxycholic acid in reducing bictegravir plasma levels. Importantly, it was recognized that treatment with ursodeoxycholic acid was no longer necessary. Furthermore, even if this therapy had still been required, alternative drugs without CYP3A4 induction potential could have been used to minimize the risk of DDI involving INIs.

A risk of failure to maintain a good virological control was also observed in four of the eight clinical cases presented here, which involved patients taking NHPs, which are frequently used by both healthy subjects and patients with various chronic conditions. In all the cases described, clinicians decided to discontinue the administration of NHPs rather than establish an appropriate interval between taking these products and antiretroviral drugs, as the clinical assessment of the patients indicated that the use of NHPs did not appear to be truly necessary. It is important to note that, although NHPs are often considered inherently safe, their use is frequently associated with DSIs that can lead to clinically relevant adverse outcomes [24,62,63,64]. PLWH are no exception in this regard [22,65,66,67]. Infectious disease specialists frequently prescribe NHPs to alleviate certain clinical conditions and the HAART-associated ADRs. For example, the prescription of red yeast rice supplements is common in cases of mild dyslipidemia [68,69,70]. Furthermore, it is not uncommon for HIV patients to take compounds containing polyvalent cations to relieve gastrointestinal symptoms [71]. In particular, Patient 8 was taking both the calcium carbonate supplement and pantoprazole at the same time for the same purpose (to alleviate gastrointestinal symptoms) and, following a medication review, the clinicians advised the patient to take only pantoprazole.

This study has several limitations, including its single-centre observational design and the relatively small sample size, and the lack of TDM. Although the Naranjo scale identified a score ranging from 2 to 5 (from ‘possible’ to ‘probable’), it remained difficult to establish causal relationships between the DDIs and DSIs identified and the AEs experienced by the patients. In fact, several factors—such as the inflammatory state, transient clinical conditions and the natural course of HIV infection—can influence the health outcome of PLWH.

In particular, with regard to the four patients who were taking NHPs containing polyvalent cations, certain unrecorded variables—such as dietary habits, the exact frequency and duration of supplementation, and the interval between taking the supplements and bictegravir—may have influenced the results.

Furthermore, only three drug interaction checkers were consulted (although these are widely used and consolidated). Such tools can help to identify contraindications or highlight the need for therapeutic and clinical monitoring or drug dose adjustment. However, it is necessary to take into account the significant differences and inconsistencies that exist between them.

Indeed, in this study, as reported in the literature [54] we found a high degree of discordance. Indeed, out of 37 interactions of major clinical relevance, in 11 cases, two out of three tools were in agreement with each other, and in only two cases, all the tools produced consistent results.

Notably, the DSI between bictegravir and polyvalent cations was identified only by Drugs.com, although it is also noted in the bictegravir SmPC.

These findings do not prove that such tools are entirely useless, but, given their inherent limitations, it seems essential to draw on multiple sources of information in order to tailor treatment as appropriately as possible. In this context, the MRDP is undoubtedly a useful strategy.

The study has several strengths. Firstly, the longitudinal design enabled us to follow the enrolled patients, obtaining a complete list of the drugs and NHPs used and recording the AEs that actually occurred, which were reported by the patients but always under the supervision of clinicians. Furthermore, although conducted at a single Italian tertiary-care centre, the high prevalence of polypharmacy revealed suggests that similar patterns of drug interactions may occur in comparable HIV outpatient populations.

## 4. Materials and Methods

### 4.1. Study Population

This longitudinal observational study is part of an ongoing project aimed at investigating genetic and non-genetic factors associated with response to the HAART. Adult patients of both sexes diagnosed with HIV infection undergoing HAART were consecutively enrolled between February 2024 and April 2026 and followed during routine outpatient visits scheduled every 3 months. The inclusion criteria were: age ≥ 18 years; confirmed HIV infection; and HAART treatment. Patients who refused to give their informed consent were excluded. The study received approval from the Campania 2 Ethics Committee (protocol no. 2024/18294) and was conducted according to the Declaration of Helsinki’s guidelines.

All patients were referred to the Infectious Diseases and Clinical Infectious Diseases Units of the San Giovanni di Dio e Ruggi d’Aragona University Hospital in Salerno. Data management was entrusted to the clinical pharmacologists of the Clinical Pharmacology Unit of the same University Hospital, in collaboration with infectious disease specialists. All participants provided their written informed consent before any study-related procedures began.

### 4.2. Data Collection and Procedures

Demographic, clinical and pharmacological data were collected for each patient, along with information on their current HAART regimen and concomitant medications, both at enrolment and during scheduled follow-up visits.

All data, including AEs related to DDIs or DSIs were recorded on a case report form (CRF) and subsequently entered into an electronic database created specifically for the study.

HAART efficacy was monitored by CD4+ T-cell counts (% or cells/μL) and viremia (copies/mL).

HAART resistance was analyzed using Sanger sequencing and Next-Generation Sequencing (NGS, Illumina, Inc., San Diego, CA, USA).

No participants were lost to follow-up during the observation period. Clinical, pharmacological, and virological variables were available for all patients included in the study; therefore, no formal missing-data imputation procedures were required. Missing information regarding some drug and supplement dosages was reported as “NA” in Table 2 and Table 3.

Standardized scales were used to evaluate medication adherence (Simplified Medication Adherence Questionnaire, SMAQ [72]), sleep quality (Pittsburgh Sleep Quality Index, PSQI [73]); and anxiety status (State-Trait Anxiety Inventory—Form Y, STAI-Y [74]).

Possible DDIs or DSIs involving ARVs and drugs or supplements taken to treat concomitant conditions were assessed for each patient using three interaction checkers (Drugs.com [30]—Drugsite Trust, Auckland, New Zealand, Lexicomp [31]—Wolters Kluwer Clinical Drug Information, Inc., Hudson, OH, USA and Liverpool HIV interactions [32]—University of Liverpool, Liverpool, UK), together with consultation of the summary of product characteristics (SmPC) and the available scientific literature. The interaction checkers apply its own scale to classify severity of AEs related to drug interactions as follows: “major”, “moderate” and “minor” by Drugs.com; “avoid combination”, “consider therapy modification”, “monitor therapy” and “no action needed” by Lexicomp; and “do not coadminister”, “potential interaction” and “potential weak interaction” by Liverpool HIV interactions.

An interaction was considered highly clinically relevant when reported as “major” by Drugs.com, or “D: Consider therapy modification” and “X: Avoid Combination” by Lexicomp, or “Do Not Coadminister” by Liverpool HIV Interactions (Table 5). Unlike Drugs.com and Lexicomp, Liverpool HIV Interactions does not detect drug interactions between co-administered drugs (other than antiviral agents) for the treatment of comorbidities.

### 4.3. Sample Size Calculation

The study sample size was calculated on the basis of the primary objective of the ongoing project (the percentage of patients experiencing at least one adverse event related to HAART). Given that approximately 90 HIV-treatment-naïve patients are admitted each year to the Infectious Diseases Clinic and Infectious Diseases Units of the University Hospital of Salerno, and that, according to the literature, HAART regimens are associated with a 35% incidence of moderate or severe adverse events, we plan to include 70 patients in the study. This number will allow us to estimate an expected proportion of patients experiencing at least one adverse event of 35%, with an α-value of 5%. A 95% confidence level was set with a *p*-value of 0.05 (5%) and a Z-score of 1.96, a power of 80% with a β of 0.20 (20%), and a dropout rate of 10%. The minimum required sample size was 74 patients.

### 4.4. Information Bias Management

To reduce information bias, all DDIs/DSIs were independently assessed using three validated interaction checkers and verified through SmPC review and literature evaluation. Selection bias was minimized through consecutive patient enrolment.

### 4.5. Statistical Analysis

Descriptive analyses were performed using SPSS (version 30). Continuous variables were expressed as mean ± SD, and categorical variables as frequencies and percentages.

## 5. Conclusions

In conclusion, our findings demonstrate that HIV patients undergoing polypharmacy will, sooner or later, require the treatment of various specialists who often deal with issues belonging exclusively to their own specific area of expertise. Clinically significant adverse outcomes due to DDIs or DSIs may occur in patients treated with HAART and non-antiretroviral drugs or NHPs. A medication review process carried out by specialists in clinical pharmacology may support the clinical decision-making process of the various healthcare providers involved in the care of PLWH.

## Figures and Tables

**Table 1:** Main characteristics of the study population.

Variables	Total (*n* = 84)
Mean age ± SD (Years)	54.6 ± 12.0
Sex (%)	
Female	17.9
Male	82.1
Ethnicity (%)	
Caucasian	91.6
African	3.8
Latin American	1.2
Cigarette Smoking (%)	
Non-Smokers	47.4
Smokers	51.3
Former Smokers	13.9
HAART regimens (%)	
Bictegravir/Tenofovir Alafenamide/Emtricitabine	40.5
Dolutegravir/Lamivudine	16.7
Darunavir/Cobicistat/Tenofovir Alafenamide/Emtricitabine	7.1
Tenofovir Alafenamide/Emtricitabine/Rilpivirine	9.5
Comorbidity (%)	
Patients with at least one comorbidity	78.6
Comorbidities (%)	
Hypertension	31
Hypercholesterolemia	29.8
Anxiety–Depressive Syndrome	8.7
Chronic Hepatitis C	8
Type II Diabetes	6
COPD	6
Number of administered drugs (Mean ± SD)	5.9 ± 2.7
Drugs co-administered with HAART (%)	
Rosuvastatin	23.8
Folic Acid	14.3
Cholecalciferol	10.7
Cotrimoxazole	8.3
Atorvastatin	8.3
Omeprazole	8.3
Pantoprazole	8.3
Bisoprolol	7.1
Ramipril	6.0
Rosuvastatin + Ezetimibe	6.0
Metformin	4.8
Ezetimibe	4.8
Alprazolam	2.5
Escitalopram	1.2
Lorazepam	1.2
Diazepam	1.2
Supplements co-administered with HAART (%)	
Patients taking at least one supplement	35.7
Type of supplements (%)	
Multivitamins	10.7
Vitamin D	8.3
Vitamin C	7.1
Melatonin	4.8
Red Yeast Rice	1.2

Abbreviations: COPD, chronic obstructive pulmonary disease; HAART, Highly Active Antiretroviral Therapy; *n*, number; SD, standard deviation.

**Table 2:** Polypharmacy of enrolled patients showing potential DDI-related AEs.

Patient	Drugs	Posology
1	Darunavir/Cobicistat	800/150 mg, once daily
	Emtricitabine/Tenofovir disoproxil	200/245 mg, once daily
	Dolutegravir	50 mg, once daily
	Amlodipine	10 mg, once daily
	Acetylsalicylic acid	100 mg, once daily
	Sotalol	80 mg, twice daily
	Apixaban	2.5 mg, twice daily
	Alprazolam	0.5 mg, once daily
	Paroxetine	20 mg, once daily
	Furosemide (a)	25 mg, 1 every two days
	Hydrochlorotiazide (b)	25 mg, once daily
	Rosuvastatin (a)	NA
	Evolocumab (b)	140 mg, 2 per months
2	Emtricitabine/Rilpivirine/Tenofovir alafenamide	200/25/25 mg, once daily
	Apixaban	5 mg, twice daily
	Furosemide	125 mg, once daily
	Bisoprolol	1.25 mg, twice daily
	Telmisartan	40 mg, once daily
	Hydroxychloroquine	200 mg, once daily
	Metolazone	5 mg, weekly
	Allopurinol	300 mg, once daily
3	Bictegravir/Emtricitabine/Tenofovir alafenamide	50/200/25 mg, once daily
	Atenolol	50 mg, once daily
	Empagliflozin	10 mg, once daily
	Rivaroxaban	20 mg, once daily
	Ezetimibe/Rosuvastatin	5/10 mg, once daily
	Amiodarone	NA
	Ursodeoxycholic acid	450 mg, once daily
4	Bictegravir/Emtricitabine/Tenofovir alafenamide	50/200/25 mg, once daily
	Nebivolol/hydrochlorothiazide	NA
	Perindopril	NA
	Lormetazepam	NA
	Paroxetine	NA
	Atorvastatin	20 mg, once daily
	Fenofibrate	145 mg, once daily
	Pantoprazole	40 mg, once daily
	Doxazosin	NA

(a) Drugs at baseline; (b) drugs after switch or swap. Abbreviations: AEs, adverse events; twice daily, bis in once daily; DDI, drug–drug interaction; NA, not available.

**Table 3:** Polypharmacy of enrolled patients showing potential DSI-related AEs.

Patient	Drugs/Supplements	Posology
5	Bictegravir/Emtricitabine/Tenofovir alafenamide	50/200/25 mg, once daily
	Atorvastatin	20 mg, once daily
	Olmesartan	NA
	Multivitamins with minerals	NA
6	Bictegravir/Emtricitabine/Tenofovir alafenamide	50/200/25 mg, once daily
	Atorvastatin	10 mg, once daily
	Fenofibrate	145 mg, once daily
	Perindopril/Bisoprolol	NA
	Multivitamins with minerals	NA
7	Bictegravir/Emtricitabine/Tenofovir alafenamide	50/200/25 mg, once daily
	Hydroxychloroquine	200 mg, once daily
	Cholecalciferol	25,000 UI
	Multivitamins with minerals	NA
8	Bictegravir/Emtricitabine/Tenofovir alafenamide	50/200/25 mg, once daily
	Nebivolol	2.5 mg, once daily
	Bempedoic acid/Ezetimibe	180/10 mg, once daily
	Pantoprazole	20 mg, once daily
	Multivitamins with minerals	NA
	NeoBianacid^®^	1.55 g, once daily

Abbreviations: AEs, adverse events; DSI, drug–supplement interaction; NA, not available.

**Table 4:** Table illustrates clinical characteristics of the eight case reports described in the text, the severity of DDIs/DSIs and the proposed mechanism, as well as clinical outcomes following MRDP.

Pt n°	SMAQ	PSQI Score	STAI-Y Score	Follow-Up	HAART Resistance	Experienced AE	DDI/DSI	DDI/DSI Proposed Mechanism	DDI/DSI Severity Grade			NA Score	Intervention Following MRDP	Clinical Outcome
									Drugs.com	Lexicomp	Liverpool HIV Int.			
1	Adherent	18	65	7 months	Absent	Drowsiness, Dyspnoea	Alprazolam—cobicistat	Cobicistat, a potent CYP3A4 inhibitor, may increase the plasma exposure of alprazolam, which is extensively metabolized by this isoenzyme.	Major	D: Consider therapy modification	Potential interaction	2, possible	Replacing alprazolam with zolpidem.	Resolution of dyspnoea. Undetectable viral load.
2	Adherent	3	20	10 months	Absent	QTc prolongation (510 ms)	Rilpivirine -hydroxychloroquine	Hydroxychloroquine, when used in combination with other drugs associated with QTc prolongation, such as rilpivirine, may increase the risk of ventricular arrhythmias.	Major	No interaction found	No interaction found	3, possible	The HAART regimen was replaced with bictegravir/emtricitabine/tenofovir alafenamide that is not associated with significant cardiac effects.	Undetectable viral load. QTc interval value unknown.
3	Adherent	NA	NA	3 months	Absent	Increased viral load (3490 copies/mL)	Bictegravir—ursodeoxycholic acid	Ursodeoxycholic acid may reduce plasma concentrations of bictegravir via a potential inducing effect on the CYP3A and UGT1A1 enzymes, which play an important role in the metabolism of bictegravir.	No interaction found	No interaction found	Potential interaction	5, probable	Discontinuation of ursodeoxycholic acid.	Viral load: 512 copies/mL.
4	Adherent	16	60	8 months	Absent	Asthenia, muscle weakness and myalgia	Atorvastatin-fenofibrate	The risk of muscle toxicity may increase if a fibrate is taken in combination with statins or other fibrates.	Major	C: Monitor therapy	No interaction found	4, possible	Lipid-lowering therapy was replaced with simvastatin/ezetimibe.	Resolution of asthenia and muscle weakness.
5	Adherent	NA	NA	10 months	Absent	Fluctuating viremias (baseline 47.8 copies/mL, first FU 95.1 copies/mL, second FU undetectable)	Bictegravir—multivitamins with minerals (magnesium hydroxide)	INIs bind to magnesium ions at the active site of the HIV integrase, reducing its binding to viral analogues and thereby compromising its antiviral efficacy.	Major	No interaction found	Potential interaction	5, probable	The multivitamin and mineral supplement has been discontinued.	Undetectable viral load.
6	Adherent	3	19	10 months	Absent	Detectable viral load (baseline undetectable, first FU < 20 copies/mL, second FU 25 copies/mL)	Bictegravir—multivitamins with minerals (magnesium hydroxide)	INIs bind to magnesium ions at the active site of the HIV integrase, reducing its binding to viral analogues and thereby compromising its antiviral efficacy.	Major	No interaction found	Potential interaction	5, probable	The multivitamin and mineral supplement has been discontinued.	Undetectable viral load.
7	Adherent	3	20	10 months	Absent	Fluctuating viremias (baseline <20 copies/mL, first FU 77.6 copies/mL, second FU undetectable)	Bictegravir—multivitamins with minerals (magnesium hydroxide)	INIs bind to magnesium ions at the active site of the HIV integrase, reducing its binding to viral analogues and thereby compromising its antiviral efficacy.	Major	No interaction found	Potential interaction	5, probable	The multivitamin and mineral supplement has been discontinued.	Undetectable viral load.
8	Adherent	4	20	8 months	Absent	Detectable viral load (baseline 59.8 copies/mL, first FU 72.5 copies/mL, second FU 136 copies/mL)	Bictegravir—multivitamins with minerals (calcium carbonate)	INIs bind to calcium ions at the active site of the HIV integrase, reducing its binding to viral analogues and thereby compromising its antiviral efficacy.	Major	No interaction found	Potential interaction	5, probable	The multivitamin and mineral supplement has been discontinued.	Viral load: <20 copies/mL

Abbreviations: AE, adverse event; DDIs/DSIs, drug–drug/drug–supplement interactions; FU, follow-up; HAART, Highly Active Antiretroviral Therapy; MRDP, medication review and deprescribing; NA, not available; NA score, Naranjo algorithm; SMAQ, Simplified Medication Adherence Questionnaire; PSQI, Pittsburgh Sleep Quality Index; STAI-Y, State-Trait Anxiety Inventory—Form Y; Pt, Patient.

**Table 5:** Identification of pharmacological interactions classified as highly clinically relevant by the three drug interaction checkers used.

	Drugs.com	Lexicomp	Liverpool HIV Interactions
Highly clinically relevant pharmacological interactions	Major	X. Avoid combination D. Consider therapy modification	Do not coadminister

## Data Availability

The original contributions presented in this study are included in the article/Supplementary Material. Further inquiries can be directed to the corresponding author.

## Supplementary Materials

The following supporting information can be downloaded at https://www.mdpi.com/article/10.3390/ph19091383/s1: Figure S1: Study Flow Diagram; Table S1: DDIs/DSIs between ARVs and non-ARV co-medications identified by the three tools Drugs.com, Lexicomp and, Liverpool HIV Interactions, and their respective severity grade; Table S2: DDIs/DSIs involving non-ARV co-medications identified by the three tools Drugs.com, Lexicomp and, Liverpool HIV Interactions, and their respective severity grade. Table S3: STROBE Statement—checklist of items that should be included in reports of observational studies.

## Abbreviations

The following abbreviations are used in this manuscript:

ADR	Adverse drug reaction
AE	Adverse event
AF	Atrial fibrillation
AIFA	Italian Medicines Agency
BDZ	Benzodiazepine
COPD	Chronic obstructive pulmonary disease
CYP	Cytochrome
DDI	Drug–drug interaction
DSI	Drug–supplement interaction
FU	Follow-up
HAART	Highly active antiretroviral therapy
HIV	Human Immunodeficiency Virus
INI	Integrase inhibitor
LLOQ	Lower limit of quantification
NA	Not available
NHP	Natural health product
NNRTI	Non-nucleoside reverse transcriptase inhibitor
PI	Protease inhibitor
PLWH	People living with HIV
PSQI	Pittsburgh sleep quality index
SMAQ	Simplified medication adherence questionnaire
SmPC	Summary of product characteristic
STAI-Y	State-trait anxiety inventory—Form Y
US	United States
