# Supplementary material for: Need for Medication Review and Deprescribing in People Living with HIV: A Longitudinal Observational Study
Source: Pharmaceuticals (Basel). 2026 Sep 1;19(9):1383. doi: 10.3390/ph19091383 (PMC13610417; doi:10.3390/ph19091383)
Supplement: Supplementary file 1 [file pharmaceuticals-19-01383-s001.zip › pharmaceuticals-4451766-supplementary.pdf]

Table S1: DDIs/DSIs between ARVs and non-ARV co-medications identified by the three tools Drugs.com, Lexicomp and, Liverpool HIV Interactions, and their respective severity grade.

ARVs and non-ARV co-medications involved in DDIs/DSIs	DDI/DSI severity grade		
	Drugs.com	Lexicomp	Liverpool HIV Interactions
*Bictegravir and aluminium hydroxide	Major	D: Consider therapy modification	No interaction found
Bictegravir and amiodarone	Moderate	No interaction found	Potential interaction
Bictegravir and atenolol	No interaction found	No interaction found	Potential interaction
Bictegravir and azithromycin	Moderate	No interaction found	No interaction found
Bictegravir and fluconazole	Moderate	No interaction found	No interaction found
Bictegravir and hydroxychloroquine	Moderate	No interaction found	Potential interaction
*Bictegravir and magnesium hydroxide	Major	D: Consider therapy modification	No interaction found
Bictegravir and metformin	Moderate	C: Monitor therapy	Potential interaction
*Bictegravir and multivitamins with minerals	Major	No interaction found	Potential interaction
*Bictegravir and sucralfate	Major	D: Consider therapy modification	Do not coadminister
Bictegravir and trimethoprim/sulfamethoxazole	No interaction found	No interaction found	Potential weak interaction
*Bictegravir and support heartburn and acid reflux/heartburn relief	Major	D: Consider therapy modification	No interaction found
Bictegravir and ursodeoxycholic acid	No interaction found	No interaction found	Potential interaction
*Bictegravir and zinc sulfate	Major	No interaction found	Potential interaction
*Cabotegravir and multivitamin with minerals	Major	No interaction found	No interaction found
*Cabotegravir and zinc sulfate	Major	No interaction found	No interaction found
Cobicistat and amlodipine	Moderate	No interaction found	Potential interaction
*Cobicistat and alprazolam	Major	D: Consider therapy modification	Potential interaction
*Cobicistat and apixaban	Major	C: Monitor therapy	Potential interaction
Cobicistat and dolutegravir	Minor	No interaction found	Potential weak interaction
*Cobicistat and doxazosin	Moderate	D: Consider therapy modification	No interaction found
*Cobicistat and doxorubicin	Major	C: Monitor therapy	No interaction found
Cobicistat and emtricitabine	Moderate	No interaction found	No interaction found
Cobicistat and paroxetine	Moderate	No interaction found	No interaction found
Cobicistat and prednisone	Moderate	B: No action needed	Potential interaction
Cobicistat and raltegravir	Moderate	No interaction found	No interaction found
Cobicistat and rosuvastatin	Moderate	B: No action needed	Potential interaction
Cobicistat and sertraline	Moderate	No interaction found	Potential weak interaction
Cobicistat and tenofovir	Moderate	C: Monitor therapy	No interaction found
Cobicistat and trimethoprim/sulfamethoxazole	No interaction found	No interaction found	Potential weak interaction
*Cobicistat and vinblastine	Major	D: Consider therapy modification	Potential interaction
*Darunavir and alprazolam	Moderate	D: Consider therapy modification	No interaction found
Darunavir and amlodipine	No interaction found	C: Monitor therapy	Potential interaction
Darunavir and apixaban	Moderate	C: Monitor therapy	No interaction found

Darunavir and dolutegravir	Minor	No interaction found	Potential weak interaction
*Darunavir and doxazosin	No interaction found	D: Consider therapy modification	No interaction found
*Darunavir and doxorubicin	Major	C: Monitor therapy	No interaction found
Darunavir and isoniazid	Moderate	No interaction found	No interaction found
Darunavir and paroxetine	Moderate	C: Monitor therapy	No interaction found
Darunavir and prednisone	Moderate	B: No action needed	Potential interaction
*Darunavir and rosuvastatin	Major	D: Consider therapy modification	Potential interaction
Darunavir and sertraline	Moderate	C: Monitor therapy	Potential weak interaction
Darunavir and tenofovir**	Moderate	No interaction found	/
Darunavir and trimethoprim/sulfamethoxazole	No interaction found	No interaction found	Potential weak interaction
Darunavir and vinblastine	Moderate	No interaction found	Potential interaction
Dolutegravir and atenolol	No interaction found	No interaction found	Potential interaction
Dolutegravir and clopidogrel	Minor	No interaction found	No interaction found
Dolutegravir and empagliflozin	Moderate	No interaction found	No interaction found
Dolutegravir and isoniazid	Moderate	B: No action needed	No interaction found
*Dolutegravir and multivitamin with minerals	Major	D: Consider therapy modification	Potential interaction
*Dolutegravir and zinc sulfate	Major	D: Consider therapy modification	Potential interaction
Emtricitabine and capecitabine	No interaction found	No interaction found	Potential weak interaction
Emtricitabine and isoniazid	Moderate	No interaction found	No interaction found
Lamivudine and trimethoprim/sulfamethoxazole	Minor	C: Monitor therapy	Potential weak interaction
Raltegravir and omeprazole	Minor	C: Monitor therapy	No interaction found
Raltegravir and rosuvastatin	No interaction found	C: Monitor therapy	No interaction found
Raltegravir and tenofovir	No interaction found	B: No action needed	No interaction found
Rilpivirine and alfuzosin	Moderate	B: No action needed	Potential weak interaction
*Rilpivirine and hydroxychloroquine	Major	No interaction found	Potential interaction
*Rilpivirine and pantoprazole	Major	X: Avoid combination	Do not coadminister
*Tenofovir and aciclovir	Major	B: No action needed	No interaction found
Tenofovir and canagliflozin	No interaction found	No interaction found	Potential interaction
Tenofovir and capecitabine	No interaction found	No interaction found	Potential weak interaction
Tenofovir and isoniazid	Moderate	No interaction found	No interaction found
*Tenofovir and mesalamine	Major	No interaction found	No interaction found
Tenofovir and metformin	Moderate	No interaction found	No interaction found
*Tenofovir and valganciclovir	Major	C: Monitor therapy	No interaction found

Abbreviations: ARVs, Antiretroviral drugs, DDIs/DSIs, drug-drug/drug-supplement interactions.

*DDI/DSI considered highly clinically significant according to the classification of severity grade of the AE-related DDIs/DSIs reported in the text.

** Liverpool HIV Interactions enables the identification of drug interactions exclusively between antiviral and non-antiviral drugs.

Table S2: DDIs/DSIs involving non-ARV co-medications identified by the three tools Drugs.com, Lexicomp and, Liverpool HIV Interactions, and their respective severity grade.

DDIs/DSIs involving non-ARV co-medications	DDI/DSI severity grade		
	Drugs.com	Lexicomp	Liverpool HIV Interactions
Acetylsalicilic acid and aluminium hydroxide	No interaction found	B: No action needed	/
Acetylsalicilic acid and amlodipine	Moderate	No interaction found	/
*Acetylsalicilic acid and apixaban	Major	D: Consider therapy modification; C: Monitor therapy	/
Acetylsalicilic acid and atenolol	Minor	No interaction found	/
Acetylsalicilic acid and bisoprolol	Minor	No interaction found	/
Acetylsalicilic acid and canagliflozin	No interaction found	C: Monitor therapy	/
Acetylsalicilic acid and carvedilol	Minor	No interaction found	/
Acetylsalicilic acid and clopidogrel	Moderate	C: Monitor therapy	/
Acetylsalicilic acid and doxazosin	Moderate	No interaction found	/
Acetylsalicilic acid and empagliflozin	No interaction found	C: Monitor therapy	/
Acetylsalicilic acid and enalapril	Moderate	No interaction found	/
*Acetylsalicilic acid and enoxaparin	Major	C: Monitor therapy	/
Acetylsalicilic acid and esomeprazole	Minor	No interaction found	/
Acetylsalicilic acid and furosemide	Moderate	C: Monitor therapy	/
Acetylsalicilic acid and irbesartan	Moderate	No interaction found	/
Acetylsalicilic acid and nebivolol	Minor	No interaction found	/
Acetylsalicilic acid and magnesium hydroxide	No interaction found	B: No action needed	/
Acetylsalicilic acid and metformin	No interaction found	C: Monitor therapy	/
Acetylsalicilic acid and metoprolol	Minor	No interaction found	/
Acetylsalicilic acid and multivitamin with minerals	No interaction found	C: Monitor therapy	/
Acetylsalicilic acid and olmesartan	Moderate	No interaction found	/
Acetylsalicilic acid and omega 3	Moderate	C: Monitor therapy	/
Acetylsalicilic acid and pantoprazole	Minor	No interaction found	/
Acetylsalicilic acid and paroxetine	No interaction found	C: Monitor therapy	/
Acetylsalicilic acid and perindopril	Moderate	C: Monitor therapy	/
Acetylsalicilic acid and ramipril	Moderate	C: Monitor therapy	/
Acetylsalicilic acid and sotalol	Minor	No interaction found	/
Acetylsalicilic acid and sulfamethoxazole	No interaction found	No interaction found	/
Acetylsalicilic acid and vitamin C	No interaction found	C: Monitor therapy	/
Albuterol and fluticasone	Minor	No interaction found	/
Albuterol and azithromycin	Moderate	C: Monitor therapy	/
Albuterol and vilanterol	Moderate	C: Monitor therapy	/
*Alfuzosin and escitalopram	Major	C: Monitor therapy	/
Alfuzosin and formoterol	Moderate	B: No action needed	/
Alfuzosin and lorazepam	Moderate	No interaction found	/
Allopurinol and furosemide	Moderate	C: Monitor therapy	/
*Allopurinol and metolazone	Major	C: Monitor therapy	/
Alprazolam and paroxetine	Moderate	C: Monitor therapy	/
Alprazolam and sotalol	Moderate	No interaction found	/
Amlodipine and aluminium hydroxide	No interaction found	C: Monitor therapy	/
Amlodipine and atenolol	Moderate	No interaction found	/
Amlodipine and bisoprolol	Moderate	No interaction found	/

Amlodipine and clopidogrel	No interaction found	C: Monitor therapy	/
Amlodipine and empagliflozin	Moderate	No interaction found	/
Amlodipine and enalapril	Minor	No interaction found	/
Amlodipine and magnesium hydroxide	No interaction found	C: Monitor therapy	/
Amlodipine multivitamin with minerals	No interaction found	No interaction found	/
Amlodipine and nebivolol	Moderate	No interaction found	/
Amlodipine and ramipril	Minor	No interaction found	/
Atenolol and amiodarone	Moderate	C: Monitor therapy	/
Atenolol and empagliflozin	Moderate	No interaction found	/
Atenolol and multivitamin with minerals	Moderate	No interaction found	/
Atorvastatin and clopidogrel	Moderate	B: No action needed	/
Atorvastatin and empagliflozin	Moderate	No interaction found	/
Atorvastatin and esomeprazole	Moderate	B: No action needed	/
Atorvastatin and ezetimibe	Moderate	No interaction found	/
*Atorvastatin and fenofibrate	Major	C: Monitor therapy	/
Atorvastatin and omeprazole	Moderate	No interaction found	/
Atorvastatin and pantoprazole	Moderate	No interaction found	/
Azithromycin and fluconazole	Moderate	C: Monitor therapy/B: No action needed	/
Azithromycin and sulfamethoxazole	Minor	No interaction found	/
Beclomethason and formoterol	Minor	B: No action needed	/
Bisoprolol and canagliflozin	Moderate	No interaction found	/
Bisoprolol and empagliflozin	Moderate	No interaction found	/
Bisoprolol and metolazone	Moderate	No interaction found	/
Bisoprolol and multivitamin with minerals	Moderate	No interaction found	/
Bleomycin and dacarbazine	Moderate	No interaction found	/
Bleomycin and doxorubicin	Moderate	No interaction found	/
Bleomycin and filgrastim	Moderate	B: No action needed	/
Canagliflozin and enoxaparin	No interaction found	C: Monitor therapy	/
Cholecalciferol and beclomethason	Moderate	No interaction found	/
Cholecalciferol and red yeast rice	Minor	No interaction found	/
Cholecalciferol and rosuvastatin	Minor	No interaction found	/
*Cholecalciferol and multivitamins with minerals	No interaction found	X: Avoid combination	/
Dacarbazine and doxorubicin	Moderate	No interaction found	/
Dacarbazine and isoniazid	Moderate	B: No action needed	/
Dapagliflozin and alfuzosin	Moderate	No interaction found	/
Dapagliflozin and amlodipine	Moderate	No interaction found	/
Dapagliflozin and ramipril	Moderate	B: No action needed	/
Diazepam and pregabalin	Moderate	C: Monitor therapy	/
*Diclofenac and sertraline	Moderate	D: Consider therapy modification	/
*Diclofenac and tenofovir	Major	D: Consider therapy modification	/
Doxazosin and canagliflozin	Moderate	No interaction found	/
Doxazosin and lormetazepam**	/	C: Monitor therapy	/
Doxazosin and nebivolol	Moderate	C: Monitor therapy	/
Doxazosin and perindopril	Moderate	No interaction found	/
Ezetimibe and aluminium hydroxide	Moderate	No interaction found	/
Ezetimibe and magnesium hydroxide	Moderate	No interaction found	/
Ezetimibe and rosuvastatin	Moderate	No interaction found	/

Filgrastim and dacarbazine	Moderate	No interaction found	/
Filgrastim and doxorubicin	Moderate	No interaction found	/
Folic acid and sulfamethoxazole	Moderate	No interaction found	/
Folic acid and trimethoprim	Moderate	No interaction found	/
Fluticasone and vilanterol	Minor	No interaction found	/
Furosemide and alprazolam	Moderate	No interaction found	/
Furosemide and bisoprolol	Moderate	No interaction found	/
Furosemide and doxazosin	Moderate	No interaction found	/
Furosemide and levothyroxine	Moderate	No interaction found	/
Furosemide and metolazone	Moderate	No interaction found	/
Furosemide and pantoprazole	Moderate	No interaction found	/
Furosemide and sotalol	Moderate	No interaction found	/
Hydrochlorotiazide and cholecalciferol	Moderate	C: Monitor therapy	/
Hydrochlorotiazide and doxazosin	Moderate	No interaction found	/
Hydrochlorotiazide and multivitamins with minerals	No interaction found	C: Monitor therapy	/
Hydrochlorotiazide and nebivolol	Moderate	No interaction found	/
Hydrochlorotiazide and paroxetine	Moderate	C: Monitor therapy	/
Hydrochlorotiazide and perindopril	Moderate	No interaction found	/
Hydrochlorotiazide and ramipril	Moderate	No interaction found	/
Hydrochlorotiazide and semaglutide	Moderate	No interaction found	/
Hydrochlorotiazide and umeclidinium	No interaction found	C: Monitor therapy	/
Hydrochlorotiazide and vilanterol	Moderate	C: Monitor therapy	/
Hydroxychloroquine and apixaban	Moderate	No interaction found	/
Hydroxychloroquine and bisoprolol	No interaction found	C: Monitor therapy	/
Insulin glargine and acetyl salicilic acid	Moderate	No interaction found	/
*Insulin glargine and canagliflozin	Moderate	D: Consider therapy modification/ C: Monitor therapy	/
Insulin glargine and irbesartan	Moderate	No interaction found	/
Insulin glargine and lixisenatide	Moderate	No interaction found	/
Insulin glargine and minocycline	No interaction found	No interaction found	/
Insulin glargine and nebivolol	Moderate	C: Monitor therapy	/
Irbesartan and canagliflozin	Moderate	C: Monitor therapy	/
Irbesartan and doxazosin	Moderate	No interaction found	/
Irbesartan and enoxaparin	Moderate	C: Monitor therapy	/
Lamivudine and trimethoprim	Minor	C: Monitor therapy	/
Lorazepam and escitalopram	Moderate	C: Monitor therapy	/
*Methadone and sodium oxybate	Major	D: Consider therapy modification	/
Nebivolol and canagliflozin	Moderate	No interaction found	/
Nebivolol and multivitamin with minerals	Moderate	No interaction found	/
Olmesartan and empagliflozin	Moderate	No interaction found	/
Omega 3 and enoxaparine	Moderate	C: Monitor therapy	/
Omeprazole and capecitabine	Moderate	C: Monitor therapy	/
Omeprazole and melatonin	Moderate	No interaction found	/
Omeprazole and multivitamin with minerals	No interaction found	C: Monitor therapy	/
*Pantoprazole and clopidogrel	Moderate	D: Consider therapy modification	/
Pantoprazole and fenofibrate	Moderate	No interaction found	/
*Pantoprazole and mesalazine	No interaction found	D: Consider therapy modification	/

Pantoprazole and metolazone	Moderate	No interaction found	/
Pantoprazole and and multivitamin with minerals	No interaction found	C: Monitor therapy	/
Paroxetine and alprazolam	Moderate	No interaction found	/
Paroxetine and apixaban	Moderate	No interaction found	/
Paroxetine and furosemide	Moderate	No interaction found	/
Paroxetine and lormetazepam**	/	C: Monitor therapy	/
Paroxetine and nebivolol	Moderate	C: Monitor therapy	/
*Paroxetine and sotalol	No interaction found	D: Consider therapy modification	/
Prednisone and isoniazid	Moderate	B: No action needed	/
Ramipril and metformin	Moderate	C: Monitor therapy	/
Rivaroxaban and amiodarone	Moderate	B: No action needed	/
Rosuvastatin and aluminium hydroxide	No interaction found	C: Monitor therapy	/
Rosuvastatin and amiodarone	Moderate	B: No action needed	/
Rosuvastatin and magnesium hydroxide	No interaction found	C: Monitor therapy	/
Rosuvastatin and sacubitril	Moderate	B: No action needed	/
Sotalol and doxazosin	Moderate	C: Monitor therapy	/
Sulfamethoxazole and albuterol	Minor	No interaction found	/
Sulfamethoxazole and azithromycin	Minor	No interaction found	/
Sulfamethoxazole and doxorubicin	Minor	No interaction found	/
Sulfamethoxazole and fluconazole	Minor	C: Monitor therapy	/
Sulfamethoxazole and isoniazid	Moderate	No interaction found	/
Trimethoprim and valganciclovir	Minor	No interaction found	/
Valsartan and dapagliflozin	Moderate	No interaction found	/
Vinblastine and bleomycin	Moderate	No interaction found	/
Vinblastine and dacarbazine	Moderate	No interaction found	/
Vinblastine and doxorubicin	Minor	No interaction found	/
Vinblastine and filgrastim	Moderate	No interaction found	/
Vinblastine and isoniazid	Moderate	No interaction found	/

Abbreviations: ARVs, Antiretroviral drugs, DDIs/DSIs, drug-drug/drug-supplement interactions. *DDI/DSI considered highly clinically significant according to the classification of severity grade of the AE-related DDIs/DSIs reported in the text.

** The drug "lormetazepam" is not listed on the Drugs.com *Drug Interaction Checker*.

/ Liverpool HIV Interactions enables the identification of drug interactions exclusively between antiviral and non-antiviral drugs.

Table S3. STROBE Statement—checklist of items that should be included in reports of observational studies

	Item No	Recommendation	Page
**Title and abstract**	1	(*a*) Indicate the study's design with a commonly used term in the title or the abstract	1
		(*b*) Provide in the abstract an informative and balanced summary of what was done and what was found	1
**Introduction**			
Background/rationale	2	Explain the scientific background and rationale for the investigation being reported	2-3
Objectives	3	State specific objectives, including any prespecified hypotheses	3
**Methods**			
Study design	4	Present key elements of study design early in the paper	18
Setting	5	Describe the setting, locations, and relevant dates, including periods of recruitment, exposure, follow-up, and data collection	18
Participants	6	(*a*) *Cohort study*—Give the eligibility criteria, and the sources and methods of selection of participants. Describe methods of follow-up	18
		(*b*) *Cohort study*—For matched studies, give matching criteria and number of exposed and unexposed	NA
Variables	7	Clearly define all outcomes, exposures, predictors, potential confounders, and effect modifiers. Give diagnostic criteria, if applicable	19
Data sources/ measurement	8*	For each variable of interest, give sources of data and details of methods of assessment (measurement). Describe comparability of assessment methods if there is more than one group	19
Bias	9	Describe any efforts to address potential sources of bias	19
Study size	10	Explain how the study size was arrived at	19
Quantitative variables	11	Explain how quantitative variables were handled in the analyses. If applicable, describe which groupings were chosen and why	18-19
Statistical methods	12	(*a*) Describe all statistical methods, including those used to control for confounding	18
		(*b*) Describe any methods used to examine subgroups and interactions	NA
		(*c*) Explain how missing data were addressed	18
		(*d*) *Cohort study*—If applicable, explain how loss to follow-up was addressed	18
		(*e*) Describe any sensitivity analyses	NA

Continued on next page

**Results**			**Page**
Participants	13*	(a) Report numbers of individuals at each stage of study—eg numbers potentially eligible, examined for eligibility, confirmed eligible, included in the study, completing follow-up, and analysed	3-15
		(b) Give reasons for non-participation at each stage	3-15
		(c) Consider use of a flow diagram	Fig. S1
Descriptive data	14*	(a) Give characteristics of study participants (eg demographic, clinical, social) and information on exposures and potential confounders	Table 1
		(b) Indicate number of participants with missing data for each variable of interest	Table 2, 3
		(c) *Cohort study*—Summarise follow-up time (eg, average and total amount)	5, Fig. 1-3
Outcome data	15*	*Cohort study*—Report numbers of outcome events or summary measures over time	3-15 Fig 1-3, Table 4
Main results	16	(*a*) Give unadjusted estimates and, if applicable, confounder-adjusted estimates and their precision (eg, 95% confidence interval). Make clear which confounders were adjusted for and why they were included	5, NA
		(*b*) Report category boundaries when continuous variables were categorized	NA
		(*c*) If relevant, consider translating estimates of relative risk into absolute risk for a meaningful time period	NA
Other analyses	17	Report other analyses done—eg analyses of subgroups and interactions, and sensitivity analyses	Table 1-3, 4 Fig. 1-3
**Discussion**			
Key results	18	Summarise key results with reference to study objectives	16-18
Limitations	19	Discuss limitations of the study, taking into account sources of potential bias or imprecision. Discuss both direction and magnitude of any potential bias	17
Interpretation	20	Give a cautious overall interpretation of results considering objectives, limitations, multiplicity of analyses, results from similar studies, and other relevant evidence	16-18
Generalisability	21	Discuss the generalisability (external validity) of the study results	18
**Other information**			
Funding	22	Give the source of funding and the role of the funders for the present study and, if applicable, for the original study on which the present article is based	20

*Give information separately for cases and controls in case-control studies and, if applicable, for exposed and unexposed groups in cohort and cross-sectional studies.

Note: An Explanation and Elaboration article discusses each checklist item and gives methodological background and published examples of transparent reporting. The STROBE checklist is best used in conjunction with this article (freely available on the Web sites of PLoS Medicine at http://www.plosmedicine.org/, Annals of Internal Medicine at http://www.annals.org/, and Epidemiology at http://www.epidem.com/). Information on the STROBE Initiative is available at www.strobe-statement.org.

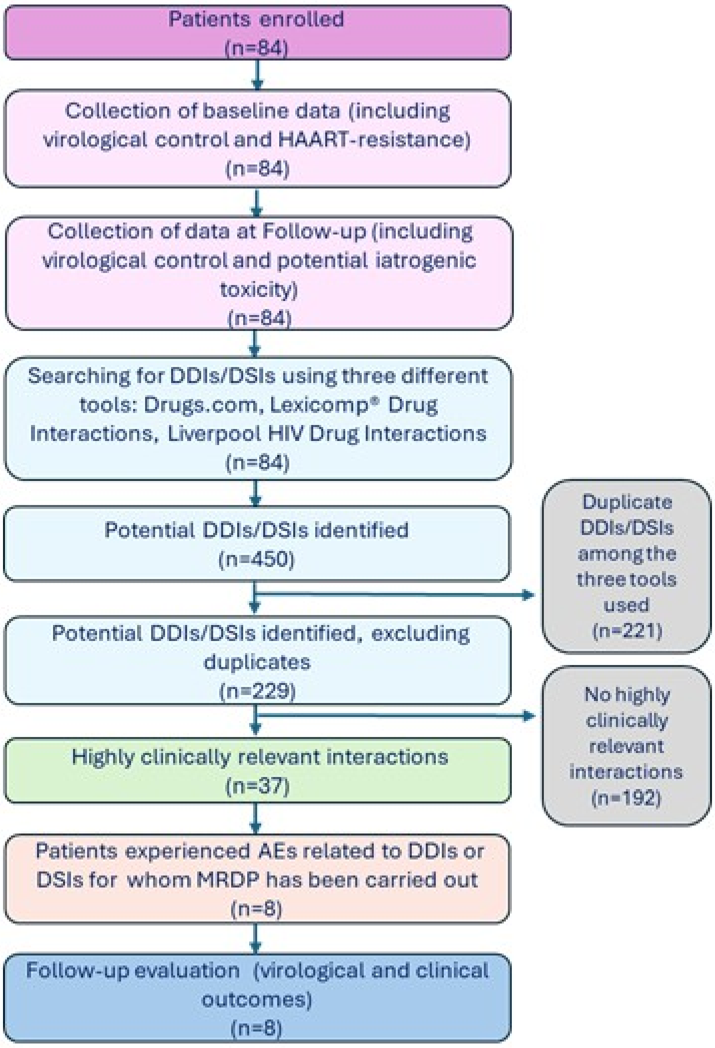

Figure S1: Study Flow Diagram.

Abbreviations: DDIs/DSIs, drug-drug/drug-supplement Interactions; HAART, Highly Active Antiretroviral Therapy; MRDP, medication review and deprescribing.
